# Development and evaluation of an index assessing adherence to the Norwegian food-based dietary guidelines: the Norwegian Dietary Guideline Index (NDGI)

**DOI:** 10.1186/s40795-024-00900-7

**Published:** 2024-07-02

**Authors:** T. H. Totland, B. Øvrebø, A. L. Brantsæter, K. Holvik, E. T. Bere, L. E. Torheim, M.H. Abel

**Affiliations:** 1 Department of Physical Health and Ageing, Division of Mental and Physical Health, Norwegian Institute of Public Health, Oslo, Norway; 2 Centre for Sustainable Diets, Norwegian Institute of Public Health, Oslo, Norway; 3 Department of Food Safety, Division of Climate and Environmental Health, Norwegian Institute of Public Health, Oslo, Norway; 4 Centre for Evaluation of Public Health Measures, Norwegian Institute of Public Health, Oslo, Norway

**Keywords:** Dietary index, Food-based dietary guidelines, Healthy eating, Nutrition

## Abstract

**Background:**

Monitoring adherence to the Norwegian food-based dietary guidelines (FBDGs) could provide valuable insight into current and future diet-related health risks. This study aimed to develop and evaluate an index measuring adherence to the Norwegian FBDGs to be used as a compact tool in nutrition surveillance suitable for inclusion in large public health surveys.

**Methods:**

The Norwegian Dietary Guideline Index (NDGI) was designed to reflect adherence to the Norwegian FBDGs on a scale from 0–100, with a higher score indicating better adherence. Dietary intakes were assessed through 19 questions, reflecting 15 dietary components covered by the Norwegian FBDGs. The NDGI was applied and evaluated using nationally representative dietary data from the cross-sectional web-based Norwegian Public Health Survey which included 8,558 adults.​

**Results:**

The population-weighted NDGI score followed a nearly normal distribution with a mean of 65 (SD 11) and range 21–99. Mean scores varied with background factors known to be associated with adherence to a healthy diet; women scored higher than men (67 vs. 64) and the score increased with age, with higher educational attainment (high 69 vs. low 64) and with better self-perceived household economy (good 67 vs. restricted 62). The NDGI captured a variety of dietary patterns that contributed to a healthy diet consistent with the FBDGs.

**Conclusion:**

The NDGI serve as a compact tool to assess and monitor adherence to the Norwegian FBDGs, to identify target groups for interventions, and to inform priorities in public health policies.​ The tool is flexible to adjustments and may be adaptable to use in other countries or settings with similar dietary guidelines.

**Supplementary Information:**

The online version contains supplementary material available at 10.1186/s40795-024-00900-7.

## Introduction

Obesity and diet-related diseases, including cardiovascular disease, type 2 diabetes, and cancer are major contributors to mortality and disability [1, 2]. Addressing modifiable behavioral risk factors, such as an unhealthy diet, is crucial for preventing these adverse health outcomes and promoting public health [3–5].

Investigating dietary patterns rather than single nutrients has proven valuable from a public health perspective by considering the complexity of nutrients and other non-nutrient substances in foods, usually eaten in combination as meals [6, 7]. As such, investigating dietary patterns in relation to disease prevention may provide opportunities for policies to focus on the combination of foods when promoting public health [7, 8]. High-quality diets, as measured by healthy eating indices, have been associated with reduced risks of all-cause mortality and several diet-related diseases [9].

Many countries have developed country-specific food-based dietary guidelines (FBDGs) as a tool to promote healthy dietary patterns [10]. These guidelines are often based on studies indicating a relationship between dietary factors and different health outcomes. The current Norwegian FBDGs, published in 2011, were developed from a synthesis of systematic reviews and evaluation of the quality of evidence of the association of foods with obesity and non-communicable diseases, and are used to set targets for public health measures and generally promote a healthy diet [11]. The Norwegian FBDGs provide the population with eleven dietary recommendations emphasizing consumption of a varied diet consisting of fruit, vegetables, whole grains, and fish; to choose lean meat, low-fat dairy foods and edible liquid oils and margarine; and to limit the intake of processed meat and red meat, foods high in salt and/or sugar and energy dense foods [12]. The English translation of the Norwegian FBDGs is presented in Supplementary table S1.

Dietary indices serve as valuable tools for assessing diet quality [7]. A-priori defined dietary indices, based on established dietary guidelines and recommendations, have advantages over data-driven approaches, as the former are based on existing knowledge, retains the complexity of food intake, and may be easier adapted by the public [13]. Dietary quality indices can be either nutrient-based, food-based, or a combination of both [7]. One example of a combined index is the American Healthy Eating Index which was designed to measure the effect of health promotion on diet [14]. Nutrient-based indices require measurement of the total diet [14]. Indices based on reported consumption of foods or food groups, such as The Australian Recommended Food Score, may be more useful as an evaluation tool of the overall diet and in epidemiologic research with less participant- and researcher burden [13]. Food-based scores are often limited to a selection of foods or food-groups and can be used to describe food patterns rather than nutrient intakes [7]. Monitoring adherence to FBDGs could provide valuable insight into diet-related health risks and indicate which of the FBDGs that may warrant attention due to low adherence, as a means for targeting preventive efforts. It may also be used as a proxy measure of overall diet quality for monitoring trends in the population [15]. Several countries have developed dietary indices based on their country-specific dietary guidelines [13, 15–20]. Given differences in available foods, consumption patterns and FBDGs, such dietary indices are bound to vary between countries [14].

In Norway, several dietary indices have been developed to measure diet quality in the general population [21–24]. However, these are not directly suitable for measuring adherence to the Norwegian FBDGs in a nutrition surveillance perspective performed as large-scale public health surveys. Two of the Norwegian indices depend on full-scale food frequency questionnaires covering the whole diet [23, 24]. The Norwegian Diet and lifestyle Index is based on the national guidelines in Norway [22], and the New Nordic Diet Score measures adherence to a healthy and environmental friendly Nordic diet [24]. However, both indices incorporate and score additional dimensions to the Norwegian FBDG. The aim of this study was to develop and evaluate an index measuring adherence to the Norwegian FBDGs to be used as a compact tool in nutrition surveillance, suitable for inclusion in large public health surveys.

## Materials and methods

### Study design

The Norwegian Public Health Survey is a cross-sectional web-based survey conducted by the Norwegian Institute of Public Health to monitor health, wellbeing, and lifestyle factors, including dietary habits [25]. In October 2020, a nationally representative sample of 26,400 adults aged ≥ 18 years was drawn from the Norwegian Population Register, representing 2,400 from each of the eleven counties in Norway. Of these, 12% did not have available contact information (by phone or e-mail) officially registered. Hence, 23,219 adults were invited to participate. The response rate was 38% (*n* = 8852), and participants who had responded to all questions for the dietary index (*n* = 8558 (97%)) were included in our study. This paper uses the Strengthening the Reporting of Observational Studies in Epidemiology – Nutritional Epidemiology (STROBE-nut) as a reporting guideline.

### Data sources and variables

The web-based questionnaire was available in Norwegian language only and included 98 questions, 33 of which were related to dietary habits. Nineteen of these were constructed to measure adherence to the Norwegian FBDGs and were combined into 15 dietary components (Table 1, Supplementary table S1). Most of the diet-related questions were phrased as food frequency questions, with specification of portion sizes, as translated into English and listed in Supplementary table S1. The participants were asked to answer the questions with the last 12 months as a reference for dietary intake.

**Table 1:** Description and rationale for scoring of the dietary components in the Norwegian dietary guideline index

Component	Description	Measure	Standard for maximum index score	Standard for minimum score of 0	Maximum index score (total 100)	Rationale
Fruit	Fruit and berries, including 1 portion of non-sweetened fruit juice	Portions/day	≥ 2.5 portions/day	0 portions/day	10	Recommended at least five portions of fruits, berries, and vegetables a day (500 g), with at least half of these from vegetables. Non-sweetened fruit juice can replace one portion of fruit (100 g). Not included potatoes, legumes, herbs, and nuts
Vegetables	Vegetables and salad, do not include potatoes	Portions/day	≥ 2.5 portions/day	0 portions/day	10	
Wholegrain	Wholegrain foods including bread, cereals, porridge, pasta, or rice	Times/day	≥ 3 times/day	0 times/day	10	Recommended intake every day corresponding to two large portions of 76–100% wholegrain foods. Max score on at least 3 times/day is given due to the large portion size and high wholegrain content suggested
Fish	Fish, whole and processed, as a lunch/dinner meal, or as sandwich topping	Portions/week	≥ 2.5 portions/ week	0 portions/ week	5	Recommended 2–3 dinner portions of fish/fish products every week, and/or preferably also to use fish as a sandwich topping (a total of 300–450 g). Six portions of sandwich toppings correspond to one dinner portion. Other seafood is not included
Red meat	Red meat, whole and processed, as a lunch/dinner meal, or as sandwich topping	Portions/week	≤ 2.5 portions/ week	≥ 7.5 portions/ week	5	Recommended a max of 2–3 dinner portions of red meat/red meat products a week, in addition to some sandwich toppings (a total of 500 g). It is assumed that six portions of sandwich toppings correspond to one dinner portion
Salt	Extra salt added at the table or during cooking, other than the original recipe/dish	rarely/never; sometimes; often, small amounts; often, moderate amounts; often, large amounts	Rarely/never	Often, large amounts	10	Recommended to limit the intake of added salt when cooking and preparing food
Fat for frying	Most used type of fat for spread	Specific products	Mainly unsaturated fat/no fat	Mainly saturated fat	5	Recommended to choose liquid or unsaturated (soft fat) for cooking and bread spread. As bread is a substantial contributor to the Norwegian diet, these are measured separately
Fat for bread	Most used type of fat for cooking	Specific products	Mainly unsaturated fat/no fat	Mainly saturated fat	5	
Cheese	Cheese, as sandwich topping or added in meals	Times/day	≤ 1 times/ day	≥ 4 times/day	5	Recommended to add low-fat dairy products at a daily basis. Intake of cheese ≥ 4 times/day gives minimum score due to high fat content
Milk/yoghurt	Milk (including yoghurt and fermented milk), non-flavored and flavored	Deciliter(dL)/day	≥ 3 to ≤ 10 dL/day	0 dL/day and ≥ 15 dL/day	10	A daily intake of low-fat dairy products is recommended, specified as three portions, with at least two portions being milk/yoghurt (assumed as 3 dL). Portion sizes differ in milk (2 dL) and yoghurt (1.25 dL). Intake of 1.5 L/day or more is considered excessive
Water	Spring or bottled water, still or carbonated, do not include flavored water	Times/day	≥ 4 times/day	0 times/day	5	Recommended to use water as a thirst-quencher Avoid sugary drinks during weekdays. Included excessive non-sweetened fruit juice consumption of more than 1 glass/day
Sugary drinks	Sugar sweetened drinks, including soft drinks, iced tea, energy drinks, fruit drinks and excess fruit juice	Times/day	0 times/day	≥ 2 times/day	5	
Chocolate/ candy	Chocolate, candy and other sweets	Times/day	0 times/day	≥ 2 times/day	5	Recommended to limit the use of sugary and energy-dense foods and drinks during weekdays
Sweet pastries	Cakes, buns, sweet biscuits and other sweet bakery goods	Times/day	0 times/day	≥ 2 times/day	5	
Salty snacks	Salty snacks including chips	Times/day	0 times/day	≥ 2 times/day	5	Recommended to limit the use of energy-dense foods and drinks during weekdays and limit the intake of added salt

Information on age, sex and county of residence were collected from the Norwegian Population Register. Educational attainment and household economy were self-reported. Education was recorded by asking for the highest completed education by four categories, and regrouped into three levels of low (by grouping primary education/primary- and lower secondary school up to 10 years and vocational education/upper secondary school/high school); medium (college/university less than 4 years); and high (college/university 4 years or more) educational attainment. Household economy was reported as self-perception of own economy, defined as how easy or difficult it is to make the money last on a day-to-day basis. The original six-category variable was grouped into three categories indicating restricted (very difficult; difficult), fair (somewhat difficult; somewhat easy) or good (easy; very easy) economy. Smoking was grouped as yes (daily or occasional) or no.

### Construction of the index

**Fig. 1:**
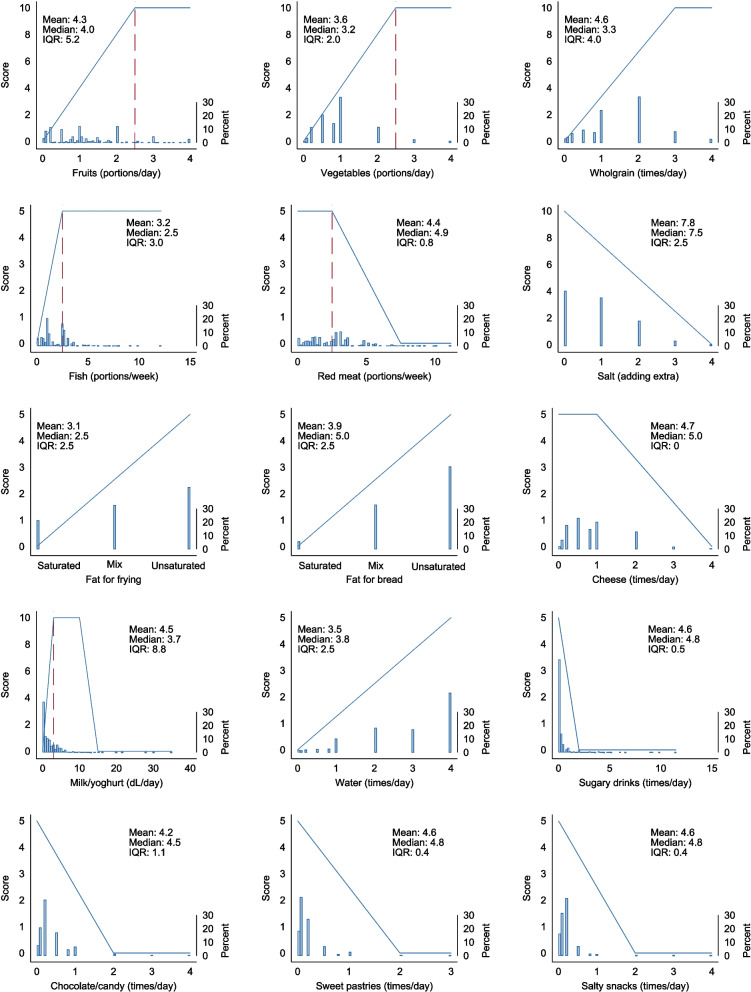
Scoring models of the NDGI for each of the dietary components (in blue lines). The histograms illustrate the population distribution of response for each component (*n* = 8,558). Dashed red lines indicate the recommended intake level that is specified for some food groups in the Norwegian food-based dietary guidelines. Mean, median and IQR are provided for each component score in the NDGI (y-axis). NDGI: Norwegian Dietary Guideline Index; IQR: Inter Quartile Range

The Norwegian Dietary Guideline Index (NDGI) was developed to assess adherence to the FBDGs distributed on a possible total score between 0 and 100 points. A higher total score indicates a better adherence to the FBDGs. The NDGI includes 15 components that reflect the intake of the following food groups which are mentioned specifically in the FBDGs: fruits (10 points), vegetables (10 points), wholegrains (10 points), fish and red meat (in total 10 points), salt (10 points), fat for bread and fat for frying (in total 10 points), cheese (5 points), milk/yoghurt (10 points), water and sugary drinks (in total 10 points), chocolate/candy and sweet pastries (in total 10 points), and salty snacks (5 points), as shown in Table 1 and Fig. 1. Some of the components were calculated based on two items in the questionnaire (fruits, fish, red meat, and milk/yoghurt).

The allocation of points to each dietary component was based on a rationale from the FBDGs when applicable, as well as a pragmatic approach considering the distribution and contribution of scores from each component in the NDGI. A consensus was reached in collaboration with nutrition researchers within the project, also considering other existing dietary indices [16, 26]. The intakes of fruits and vegetables were allocated as separate components each yielding up to 10 points. This aligns with the qualitative advice to include fruits and vegetables in every meal and is consistent with other dietary indices [15, 16, 26]. Fish and red meat were considered as protein sources, and were therefore weighted together, receiving 5 points each, as also suggested by other indices [15, 16, 26]. Adherence to recommendations related to specific nutrient-based food groups (such as fats, sodium/salt and sugar) were indirectly estimated based on the intake of specific food sources, including oils, butter, salty snacks, sugary drinks, and sweet pastries. Salty snacks and cheese were allocated 5 points each since these products represent subgroups in the FBDGs. Salty snacks contribute to the intake of energy-dense foods and added salt. Additionally, most cheeses sold in Norway have a high fat content [27], and together with red meat and butter/margarines, cheese represents the largest contributor to the intake of saturated fat in the overall diet [28].

Since not all advice in the FBDGs is complemented with specific recommended amounts, a cut-off value for compliance with the guidelines could not be defined for all components. For recommendations where quantitative measures were not available, points were allocated for minimum and maximum scores based on available information and additional qualitative advice from the FBDGs, as described in the rationale of Table 1.

### Statistical methods

Analyses were conducted using Stata SE version 16.0 (Stata Corp., College Station, TX). All analyses were population-weighted to standardize the results to the age-, sex- and county distribution of the Norwegian population aged 18 years and older in 2020, as obtained from tables published by Statistics Norway (www.ssb.no/en).

The NDGI was calculated based on data from the Norwegian Public Health Survey, summarizing the individual component scores. The distribution of the NDGI summary score was assessed for skewness and kurtosis to evaluate its deviation from normality by using the commands *summarize* and *sktest* in Stata. The differences in mean NDGI score across characteristics known to be associated with a healthy diet, such as age, sex, education, household economy and smoking, were estimated by linear regression, adjusting for covariates if relevant. There were few missing values in the dataset, as *n* = 8,558 of the total sample of *n* = 8,852 (97%) had complete data for the NDGI. Some participants had missing values for smoking (*n* = 37). For educational attainment, only participants aged > 25 years were included in the analyses (*n* = 7,855) as younger participants to a larger extent may not have attained their final educational level. We also divided the study population in five groups by quintiles of total NDGI score to explore the background characteristics and consumption of dietary components by group. The latter evaluates the index by investigating if it varies according to known differences in dietary patters, e.g., women in general have a healthier diet compared to men [16, 17, 26]. Differences between groups were tested by logistic regression for dichotomous variables, ordinal regression if variable categories had a relative ordering, and linear regression for continuous variables. A p-value < 0.05 was considered statistically significant.

For further evaluation of the index, the association between the 15 components included in the index score and each components’ association with the total index score, were assessed with pairwise Pearson correlations. Principal component analysis and a scree plot were used to identify underlying dimensions in the index score. Cronbach's alpha was calculated to explore the internal consistency of the components included in the NDGI score.

### Ethics

The Norwegian Public Health Survey (2020) was carried out under Sect. 7 of the Public Health Act, which states that municipalities, counties and the state have responsibility for promoting public health in the local communities [29]. Participant consent was given through the national identification system as the first step in completing the web-based questionnaire. The Norwegian Institute of Public Health manages the data used in this study in accordance with The Personal Data Act and The Health Research Act (§ 7) in Norway, and the General Data Protection Regulation (GDPR) in the EU. As the purpose of this work was methodological development, no further ethical approval was required.

## Results

Participants in The Norwegian Public Health Survey 2020 who were included in this study (*n* = 8,558) had a mean age of 50.8 years (SD 16.2) ranging from 18 to 92 years. Women comprised 53% of the study population and 86% reported not smoking. The distribution of educational attainment was 40% low, 22% medium and 29% high, and the self-perceived household economy was 15% restricted, 28% fair and 57% good, as shown in Supplementary table S2.

### Application of the index and variance by background factors

**Fig. 2:**
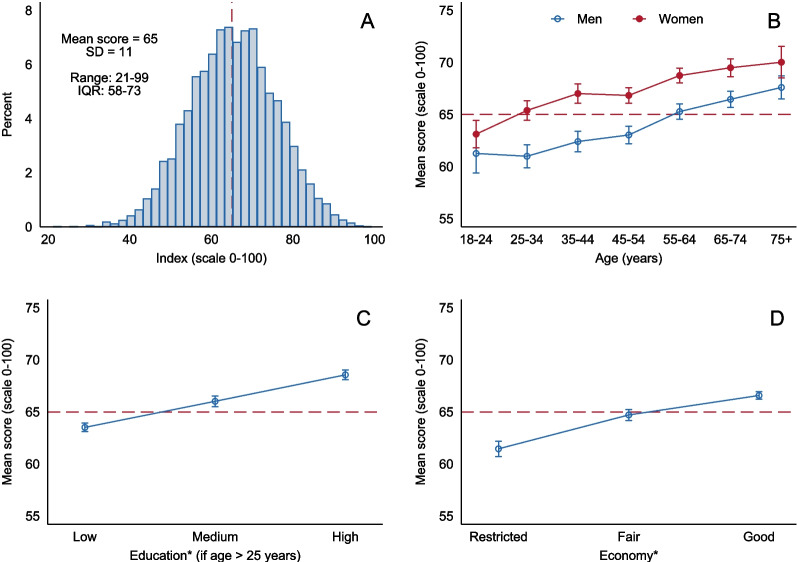
Population-weighted^1^ NDGI score in the Norwegian Public Health Survey 2020 (*n* = 8,558), overall and by background characteristics. Presented as A) distribution in the total sample, B) mean score (95% CI) by sex and age categories, C) by self-reported educational attainment and D) by self-perceived household economy. *Adjusted for sex and age. Dashed red line represents the overall mean NDGI score in the study population. ^1^The estimates were calculated using population weights weighing for county, age (5 categories) and sex in the Norwegian Public Health Survey. NDGI: Norwegian Dietary Guideline Index

Figure 2A shows the population-weighted distribution of the NDGI score, where the mean NDGI score was 65.3 (SD 10.6). The index displayed a close to normal distribution with a small skew to the left (skewness coefficient: -0.21, *p* < 0.001) and a peakedness that was not significantly different from a normal distribution (kurtosis: 3.0, *p* = 0.21). Women scored higher than men (mean 67.2 vs. 63.5), and the score increased with age in both sexes (both *p* < 0.001, Fig. 2B). Mean NDGI score increased with higher education (63.5 vs. 68.6 in low vs. high) and better self-perceived household economy (61.5 vs. 66.6 in restricted vs. good) (both: *p* < 0.001, Figs. 2C-D). More details can be found in Supplementary table S2.

Background characteristics of the study population by quintiles of NDGI scores are presented in Table 2A. The mean age, proportion of women, prevalence of non-smoking, educational attainment, and self-perceived household economy increased with higher quintiles of NDGI scores. The consumption of each dietary component by quintiles of NDGI score are shown in Table 2B. Consumption increased significantly with higher quintiles of NDGI score for fruits, vegetables, wholegrains, fish, cheese, milk/yoghurt and water. Additionally, participants in the higher quintiles were less likely to add extra salt when preparing foods and to use of saturated fats for frying and as bread spread. Consumption also decreased with higher quintiles for red meat, sugary drinks, chocolate/candy and salty snacks. The consumption of sweet pastries did not vary significantly across quintiles of NDGI scores.

**Table 2:** Population-weighted^a^ mean estimated NDGI scores by quintiles of A) Background characteristics, and B) Dietary components

	Estimate	Total adult population	1st quintile (score < 56.3)	2nd quintile	3rd quintile	4th quintile	5th quintile (score > 74.4)	*p*-value^b^
Healthy eating index score	Mean (SD)	65 (11)	50	60	65	71	80	-
**A) Background characteristics**								
Men	%	50	62	55	50	47	38	< 0.001
Women	%	50	38	45	50	53	62	< 0.001
Age, years	Mean (SD)	48 (18)	42	46	49	51	51	< 0.001
Smoking	%	14	23	17	14	11	7	< 0.001
Education								< 0.001
Low	%	47	61	49	48	41	34	
Medium	%	24	22	27	23	26	24	
High	%	29	17	24	29	33	42	
Household economy								< 0.001
Restricted	%	16	26	18	14	11	9	
Fair	%	28	29	28	30	27	24	
Good	%	57	45	54	56	62	67	
**B) Dietary components**								
Fruit, portions/day	Mean (SD)	1.1 (0.9)	0.5	0.7	1.0	1.3	2.0	< 0.001
Vegetables, portions/day	Mean (SD)	0.9 (0.7)	0.5	0.7	0.9	1.0	1.5	< 0.001
Wholegrain, times/day	Mean (SD)	1.4 (0.9)	0.7	1.1	1.4	1.7	2.1	< 0.001
Fish, portions/week	Mean (SD)	1.8 (1.2)	1.0	1.5	1.9	2.1	2.6	< 0.001
Red meat, portions/week	Mean (SD)	2.5 (1.6)	2.8	2.6	2.4	2.3	2.1	< 0.001
Salt								< 0.001
Seldom/never	%	41	22	34	40	49	58	
Sometimes	%	36	33	38	40	37	31	
Often/usually – a little	%	19	31	23	18	12	10	
Often/usually – some	%	3.8	9.5	4.8	2.4	1.7	0.6	
Often/usually – a lot	%	1.1	3.9	0.5	0.4	0.5	0.1	
Fat for frying								< 0.001
Mostly saturated fats	%	21	37	27	21	15	6.6	
Mix of fats	%	33	36	35	35	32	26	
Mostly unsaturated fats	%	46	27	38	44	54	67	
Fat for bread								< 0.001
Mostly saturated fats	%	5.8	10	5.8	6.0	3.9	2.6	
Mix of fats	%	33	42	37	34	29	24	
Mostly unsaturated fats	%	61	47	57	61	67	74	
Cheese, times/day	Mean (SD)	0.8 (0.7)	0.7	0.7	0.8	0.9	0.9	< 0.001
Milk/yoghurt, dL/day	Mean (SD)	1.8 (2.2)	0.8	1.3	1.7	2.2	3.0	< 0.001
Water, times/day	Mean (SD)	2.8 (1.3)	2.0	2.6	2.9	3.0	3.4	< 0.001
Sugary drinks, times/day	Mean (SD)	0.3 (0.7)	0.5	0.3	0.2	0.1	0.1	< 0.001
Chocolate/candy, times/day	Mean (SD)	0.3 (0.4)	0.4	0.3	0.3	0.3	0.3	< 0.001
Sweet pastries, times/day	Mean (SD)	0.2 (0.2)	0.2	0.2	0.2	0.2	0.2	0.78
Salty snacks, times/day	Mean (SD)	0.2 (0.2)	0.2	0.2	0.2	0.2	0.1	< 0.001

*NDGI* Norwegian Dietary Guideline Index, *SD* standard deviation

^a^The estimates were calculated by using population weights weighing for county, age (5 categories) and sex in the Norwegian Public Health Survey

^b^*p*-values were calculated by using logistic regression for dichotomous variables, linear regression for continuous variables, and chi-square test for categorical variables

### Composition of the index

The NDGI scoring model assigns a continuous scale between 0–10 or 0–5 points to each dietary component. Figure 1 illustrates population-weighted distributions in response for each component. The components contributed with a median score between 2.5–5.0 to the total index score, except for salt, which had a median score of 7.5. The components fruit, vegetables, wholegrain and milk/yoghurt displayed the largest deviations from the recommendations with a median NDGI score < 50% of the possible maximum component score (Fig. 1).

The highest correlation coefficient between single components in the NDGI was observed for intake of fruits and vegetables (*r* = 0.39) (Supplementary table S3). Correlations between each component and the total NDGI score ranged from -0.06 to 0.59. The components with the highest correlation coefficient to the total NDGI score, were fruits (*r* = 0.59), wholegrains (*r* = 0.58), and vegetables (*r* = 0.53). Cheese (*r* = -0.06), sweet pastries (*r* = 0.03) and chocolate/candy (*r* = 0.16) were the components with the lowest correlation to total NDGI score (Supplementary table S3).

**Fig. 3:**
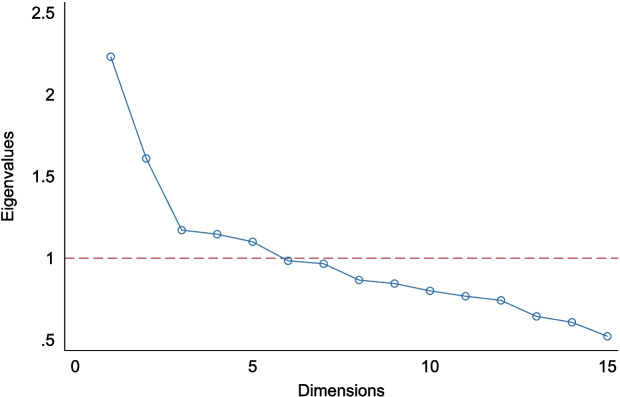
Scree plot of dimensionalities from principal component analysis of the Norwegian Dietary Guideline Index

Variation in NDGI score was explained by multiple dimensions, with five dimensions having an eigenvalue above one (Fig. 3, Supplementary table S4). The first three dimensions accounted for most of the total variance. Specifically, the first dimension explained 15%, the second explained 11% and the third 8%, corresponding to cumulative 33% (Supplementary table S4). The first dimension primarily reflected variation in components recommended for increased intake in the Norwegian FBDGs, such as fruits, vegetables, wholegrains, fish, and water. The second pattern captured variation in components where limiting intake is recommended in the Norwegian FBDGs, such as sugary drinks, chocolate/candy, sweet pastries, and salty snacks. The third dimension was associated with variation in red meat, cheese, fat for frying and fat on bread, representing a more traditional food pattern (Supplementary table S5). The internal consistency between the components in the NDGI was low (Cronbach’s alpha of 0.50).

Data is extracted from the Norwegian Public Health Survey (*n* = 8558).

## Discussion

We found that the NDGI captured variance in adherence to dietary guidelines on a continuous scale. Hence, it represents a suitable tool for monitoring trends in adherence to the Norwegian FBDGs. It also has the potential to identify dietary challenges and specific subgroups of importance, which altogether may help guide priorities in public health policies.

The mean score of 65 out of 100 (range 21–99), where 100 would represent a diet adhering to the Norwegian FBDGs, suggests that the adult population of Norway has a potential of improved adherence to the FBDGs. Results from the latest nationally representative dietary survey among adults in Norway from 2010–11 indicated that each of the FBDGs was achieved by between 15 to 45% of men and 13 to 67% of women [28]. Vegetable intake accounted for the lowest percentage and red meat intake to the highest percentage of adherence in both sexes. In the same population, between 5 and 65% had high adherence to each of 12 dietary components as reflected in the FBDGs according to the Norwegian Diet Index [22].

The specific FBDGs differ somewhat between countries, and dietary indices across countries do not cover all the same aspects of dietary recommendations and FBDGs [14]. Findings in studies using different dietary indices are therefore not directly comparable. However, similar adherence to the FBDGs measured through dietary quality indices has been described in previous population studies from Australia and Denmark [16, 17]. A score closer to half-way adherence was observed in other European countries [18, 30], the United States [31], and more recently in Vietnam [20]. In the United States, a decrease in diet quality between 2011–2018 in adults was shown using a FBDG index, with a significant change in score from 55% in 2011–2012 to 53% in 2017–2018 [31].

The distribution of sociodemographic characteristics across quintiles of NDGI scores shows the ability of the index to capture population variation in diet quality. Overall, women, and participants of older age, higher education and better self-perceived household economy, had better adherence to the Norwegian FBDGs. This is expected and consistent with findings from testing other food-based indices [16–18, 20, 30] or a combination of nutrient- and food-based indices [19, 26]. Better adherence to the FBDGs in women has also been reported in other countries [16, 17, 26]. The association between higher age and adherence to FBDGs in our sample has consistently been reported in other Norwegian representative samples [28, 32] as well as in population-based surveys in other countries [16–19, 26]. Older individuals may follow FBDGs to a larger extent as they may be more health conscious and independent in old age [33]. In Norway, older generations tend to eat more traditional dishes rather than dishes of different origins, eat less fast foods and in restaurants than younger individuals, although they more often eat out in shopping centers and cafes than middle aged adults [34]. Higher education [19, 28] and better socioeconomic position [19] have consistently been associated with a better adherence to the FBDGs in Scandinavian studies, as in other parts of the world [16, 20]. However, two studies from the Netherlands [30] and Spain [18] did not find an association between adherence to the dietary guidelines and education.

Smoking was used as a proxy to whether the NDGI was able to detect differences in scores by health behavior between groups of people with known differences in diet quality. Better adherence to the FBDGs was found for non-smokers, which agrees with findings in other studies [18, 26, 30].

### Methodological considerations

The NDGI was developed to evaluate adherence of habitual dietary intake to the Norwegian FBDGs. The implementation of an index measuring adherence to Norwegian FBDGs is of importance in a nutrition surveillance perspective. The tool is based on a short food frequency questionnaire with 19 items. In comparison, the Norwegian Diet Index, recently published by the University of Oslo, is aiming to measure a healthy diet and a healthy lifestyle in line with the national guidelines in Norway [22]. However, its practicality as a nutrition surveillance tool in large-scale public health surveys may be limited due to its requirement for more detailed information. Yet another index has been developed to measure adherence to a New Nordic Diet, measuring adherence to an environmentally sustainable and healthy Nordic diet rather than the Norwegian FBDGs [24]. The NDGI is developed with the purpose to measure adherence to the Norwegian FBDGs as communicated by the Norwegian Directorate of Health [12], with the possibility to be carried out in a frequent manner in larger population groups. The use of the NDGI is based on the proposed short food frequency questionnaire but is also feasible to apply on more comprehensive data sources, such as larger food frequency questionnaires, 24 h recalls and food records, provided that data on the specific components are included.

Each component in the NDGI was given a standard for maximum and minimum score, based on a rationale derived from quantitative and qualitative FBDGs where applicable. Intake frequency in the population was also considered, to ensure distribution of scores in the total NDGI score. Similar to the Healthy Eating Index [26], the index gave a maximum of 60 points (out of 100) to components to eat in adequacy, and 40 points allocated to components to be consumed in moderation. The level of detail between the two indices differs extensively, as the Healthy Eating Index is based on a much more comprehensive tool, measuring alignment of nutrients in addition to the FBDGs. In contrast to the Healthy Eating Index, the NDGI does not capture all aspects such as the fat content in meat and dairy products.

The median population-weighted score for each component in the NDGI shows that all have potential to improve the NDGI score. Using principal component analysis, we found the index to be multidimensional and predominantly three patterns explained the most variance in scores. These findings indicate that dietary behavior in accordance with the FBDGs is not a unidimensional construct where all factors that contribute to a healthy diet are strongly correlated. Rather, the score captures several dimensions or dietary patterns. This corresponds with the findings for evaluation of the American Healthy Eating Index [26].

The intercorrelations between components in the NDGI score were between -0.06 to 0.59, and the Cronbach’s alpha was 0.50 indicating a low to moderate internal consistency of the components in the score. This agrees with the finding of multidimensionality, as all people do not necessarily meet all the same aspects of the FBDGs. As mentioned by Reedy et al. when an index is multidimensional, captures a full diet, and is evaluated in an entire population, a lower Cronbach’s alpha is expected [26].

### Implications

The NDGI is calculated based on a compact tool that is suitable for being incorporated in large public health surveys. Hence, it may aid in following time trends in adherence to the FBDGs in the adult population and subgroups living in Norway, by synthesizing several dietary components into one overall score. The NDGI can be used in nutrition surveillance for monitoring trends in food choices over time and may thus provide a basis for planning, prioritizing, and targeting public health policy aimed to improve diet, also in subgroups of age, sex, and socioeconomic factors. Additionally, the NDGI can be used as an indicator of diet quality when studying associations between lifestyle factors and health outcomes. The NDGI is flexible and could be adapted to updated country specific guidelines and may also be an applicable instrument in other countries or settings with comparable FBDGs.

When applying the NDGI in the current sample, we observed that men and individuals with low education had the greatest potential for improving their diet to correspond with the FBDGs. Furthermore, the FBDGs covering fruit, vegetables, wholegrain and dairy (milk/yoghurt) obtained the lowest relative median component scores of adherences and hold a large potential for improvement. Thus, these FBDGs should be given more attention when trying to improve adherence to the FBDGs in the population. Further studies are required to validate the dietary questions included in the NDGI, and to investigate associations between the NDGI and health outcomes in longitudinal studies.

### Strengths and limitations

Although the NDGI does not capture all aspects in the FBDGs, it provides a continuous score suitable for capturing important parts of the dietary complexity and adherence to the Norwegian FBDGs in a relatively simple way. With reasonable participation and few missing values, this may indicate a feasible tool to be used in larger public health surveys. We consider this as a strength and to be of interest to public health researchers and to policy- and decision makers.

A limitation of the study is that dietary intake was self-reported, which may introduce bias either consciously or unconsciously. The use of a food frequency questionnaire relies on the self-report of data retrospectively, which may be affected by the ability to accurately recall information. Another limitation is the lack of knowledge about the degree to which the questionnaire reflects the actual diet in the population. A study of the relative validity of these dietary questions is planned in the ongoing national dietary survey among adults in Norway. The lack of additional details about dietary intake from the food frequency questionnaire hindered us from evaluating some of the specific recommendations in the FBDGs such as portion sizes and specific advice on food quality, such as the content of fat in meat and dairy products. As some of the FBDGs do not include quantitative measures, a pragmatic and partly data driven approach was used to include both quantitative and qualitative guidelines in the weighing of components, which may have influenced the scoring of the NDGI.

The study population was limited to adults, and thus there is a lack of generalizability to children and adolescents. The National Public Health Survey had a 38% participation rate. Of the individuals who were unreachable by phone or e-mail, 56% were aged above 75 years, and the web-based questionnaire was probably not adequate to capture the variance in diet in the oldest part of the population in Norway. Self-reported education and self-perceived household economy may not necessarily reflect the equivalent data in official registries but did still capture variance in adherence in the diet. Additionally, the design of the study may introduce healthy volunteer bias. Notwithstanding these limitations, our study was based on a representative sample of adults living in Norway.

The scoring of components was made on a continuous scale between minimum and maximum cut-off points, which allows for preserving the statistical power of the data. However, the score did not account for food habits other than the FBDGs, such as with a restricted diet or alternative dietary regimes. Despite the lack of details in the scoring of components, measurement errors are expected to be consistent over time, and the NDGI may as such be suitable to monitor adherence to FBDG if repeated over time. The NDGI can be used in nutrition surveillance as a diet quality tool to complement the more extensive national dietary surveys.

## Conclusion

The NDGI is a compact tool that can be incorporated into larger public health surveys and provides the opportunity to measure and track adherence to the Norwegian FBDGs in the population. The tool is flexible to adjustments and may be adapted to revisions or settings with similar dietary guidelines. Our study demonstrates that this tool may be suitable to evaluate adherence to the Norwegian FBDGs, and thereby assist to identify specific target groups and dietary challenges in nutrition surveillance.

### Supplementary Information

Supplementary Material 1.

## Data Availability

The dataset generated and/or analyzed during the current study is not publicly available due to data protection regulations, but supplementary information files are added for transparency of the data. Data collection tools are available in Supplementary table S1. The corresponding author may be contacted for clarification on reasonable request.

## Abbreviations

FBDGs: Food-based dietary guidelines
NDGI: Norwegian dietary guideline index
