# Supplementary material for: Development and evaluation of an index assessing adherence to the Norwegian food-based dietary guidelines: the Norwegian Dietary Guideline Index (NDGI)
Source: BMC Nutr. 2024 Jul 2;10:94. doi: 10.1186/s40795-024-00900-7 (PMC11218056; doi:10.1186/s40795-024-00900-7)
Supplement: Supplementary file 1 — Supplementary Material 1. [file 40795_2024_900_MOESM1_ESM.rtf]

Supplementary table S1
Table S1. Food-based dietary guidelines Norwegian Directorate of Health. Food Based Dietary Guidelines and Nutrition Recommendations [Kostrådene og næringsstoffer] [Web Page] Oslo, Norway: Norwegian Directorate of Health; 2016 [updated 24/10-2022; cited 2022 7/10]. Available from: https://www.helsedirektoratet.no/faglige-rad/kostradene-og-naeringsstoffer. and questions from the survey included in the Norwegian Dietary Guideline Index.
Norwegian FBDG	Questions in the Norwegian Public Health Survey including answer options	Component in the index	
Eat at least five portions of vegetables, fruit and berries every day.  	-	Approximately how much fruits and berries do you eat?
(Rarely/never; 1-3 times per month; 1-2 times per week; 3-4 times per week; 5-6 times per week; 1 time per day; 2 times daily; 3 times daily; More than 3 times a day)

-	Approximately how much vegetables do you eat? 
(Rarely/never; 1-3 times per month; 1-2 times per week; 3-4 times per week; 5-6 times per week; 1 time per day; 2 times daily; 3 times daily; More than 3 times a day)

-	How often do you drink juice/fruit smoothie? 
(Rarely/never; 1-3 times per month; 1-2 times per week; 3-4 times per week; 5-6 times per week; 1 time per day; 2 times daily; 3 times daily; More than 3 times a day)
-	Approximately how much juice/fruit smoothie do you drink in total per day/week?
(Provide approximate number of dL (one glass = 2 dL))	Fruit


Vegetables


Fruit
Sugary drinks	
Eat wholegrain foods every day.  	-	Approximately how often do you eat wholegrain products (e.g. bread, muesli, oatmeal, wholegrain pasta)? Think back over the last 12 months when you respond. Whole wheat bread such as 'kneipp' bread or whole wheat bread marked with at least 50% whole grain content on the "Bread Scale".
(Rarely/never; 1-3 times per month; 1-2 times per week; 3-4 times per week; 5-6 times per week; 1 time per day; 2 times daily; 3 times daily; More than 3 times a day)	Wholegrain 	
Eat fish two to three times a week. You can also use fish as a sandwich topping.  	-	Approximately how often do you eat fish as a main course for dinner or lunch?
(Rarely/never; 1-3 times per month; 1 time per week; 2-3 times per week; 4-5 times per week; 6-7 times per week; Several times per day)
-	Approximately how often do you eat fish as toppings on one slice of bread (e.g., caviar, mackerel in tomato, tuna salad, fishcakes)?
(Rarely/never; 1-3 times per month; 1-2 times per week; 3-4 times per week; 5-6 times per week; 1 time per day; 2 times daily; 3 times daily; More than 3 times a day)	Fish	
Choose lean meat and lean meat products. Limit the amount of processed meat and red meat.  	-	Approximately how often do you eat red meat as the main course for dinner or lunch (excluding toppings)? Red meat is meat from pork, cattle (cow/bull/calf), sheep and goat. Meat from chicken or turkey is not red meat.
(Rarely/never; 1-3 times per month; 1 time per week; 2-3 times per week; 4-5 times per week; 6-7 times per week; Several times per day)
-	Approximately how often do you eat red meat as toppings on one slice of bread (e.g., ham, salami, liver pâte)? Red meat is meat from pork, cattle (cow/bull/calf), sheep and goat. Meat from chicken or turkey is not red meat.
(Rarely/never; 1-3 times per month; 1-2 times per week; 3-4 times per week; 5-6 times per week; 1 time per day; 2 times daily; 3 times daily; More than 3 times a day)	Red meat	
Include low-fat dairy foods in your daily diet.  	-	Approximately how often do you eat cheese as a topping on one slice of bread or in a dinner dish (e.g., pizza, lasagna, salad and tacos)?
(Rarely/never; 1-3 times per month; 1-2 times per week; 3-4 times per week; 5-6 times per week; 1 time per day; 2 times daily; 3 times daily; More than 3 times a day)

-	How often do you drink cow's milk/fermented cow's milk (non-flavored or flavored, e.g., fruit/berries, cocoa, coffee)? 
(Rarely/never; 1-3 times per month; 1-2 times per week; 3-4 times per week; 5-6 times per week; 1 time per day; 2 times daily; 3 times daily; More than 3 times a day)
-	Approximately how much cow's milk/fermented cow's milk do you drink in total per week? 
(Provide approximate number of dL (one glass = 2 dL))
-	Approximately how much yogurt do you eat? Answer in the number of portions where a portion corresponds to about 1.5 dL.
(Rarely/never; 1-3 portions per month; 1-2 portions per week; 3-4 portions per week; 5-6 portions per week; 1 portion daily; 2 portions daily; 3 portions daily; More than 3 portions daily)	Cheese


Milk/yoghurt


	
Choose edible liquid oils, liquid margarine and soft margarine spreads instead of hard margarines and butter.  	-	Fat for frying – what do you use most often?
(Dairy butter; Bremykt; Hard margarin (e.g., Melange); Smøremyk margarine (e.g., Soft, Vita Olivero); Liquid margarine (e.g., Liquid Bremykt, Liquid Olivero with butter and olive oil); Liquid plant oil(s) (e.g., olive, rapeseed, soybean); Coconut oil; Other; Don't use this; Don't know)

-	Fat on bread/crispbread – what do you use most often?
(Dairy butter; Bremykt; Brelett; Hard margarine (e.g., Melange); Smøremyk margarine (e.g., Soft, Vita Olivero); Light margarine (other than Brelett); Mayonnaise/oil; Coconut oil; Other; Don't use this; Don't know)	Fat for frying


Fat for bread	
Choose foods that are low in salt and limit the use of salt when preparing food and eating.  	-	When preparing food at home, do you usually add some extra salt when preparing or eating the meal (beyond what is included in a recipe or in the ready meal)?
(Rarely/never; Sometimes; Often, small amount; Often, moderate amount; Often, large amount)

-	Approximately how often do you eat crisps and salty snacks? 
(Rarely/never; 1-3 times per month; 1-2 times per week; 3-4 times per week; 5-6 times per week; 1 time per day; 2 times daily; 3 times daily; More than 3 times a day)	Salt


Salty snacks	
Avoid foods and drinks that are high in sugar. 	-	Approximately how often do you eat chocolate and other candy?
(Rarely/never; 1-3 times per month; 1-2 times per week; 3-4 times per week; 5-6 times per week; 1 time per day; 2 times daily; 3 times daily; More than 3 times a day)
	
-	Approximately how often do you eat cakes/buns/sweet biscuits?	
(Rarely/never; 1-3 times per month; 1-2 times per week; 3-4 times per week; 5-6 times per week; 1 time per day; 2 times daily; 3 times daily; More than 3 times a day)	Chocolate/candy


Sweet pastries

	
Choose water as a thirst-quencher. Avoid sugary drinks during weekdays. 	-	How often do you drink water? 
(Rarely/never; 1-3 times per month; 1-2 times per week; 3-4 times per week; 5-6 times per week; 1 time per day; 2 times daily; 3 times daily; More than 3 times a day)

-	How often do you drink sugary soft drinks/juices/soft drinks (including iced tea, energy drinks, sports drinks, nectar)?
(Rarely/never; 1-3 times per month; 1-2 times per week; 3-4 times per week; 5-6 times per week; 1 time per day; 2 times daily; 3 times daily; More than 3 times a day)	Water


Sugary drinks
	
Enjoy a varied diet with lots of vegetables, fruit and berries, wholegrain foods and fish, and limited amounts of processed meat, red meat, salt and sugar.  	This is covered by the questions above.		
Maintain a good balance between the amount of energy you obtain through food and drinks and the amount of energy you expend through physical activity.  	This recommendation is the rationale for including some of the components with no specific FBDG in the index, such as sugary drinks, chocolate/candy, salty snacks, and sweet pastries. It is recommended to limit the consumption of foods contributing with excess calories per unit of food. Measures of physical activity is not covered by the Norwegian Dietary Guideline Index
		
dL: deciliter; FBDG: food-based dietary guideline	
Supplementary table S2
Table S2. Background characteristics (sex, age, education, and perceived household economy) in the Norwegian Public Health Survey study sample with estimated population-weighted mean (95% confidence intervals) of the Norwegian Dietary Guidelines Index (NDGI)
Background characteristics	Study sample
n (%)	Population-weighted1 NDGI score 
mean (95% CI)	p-value2	
Total population (≥18 years)	8558 (100)	65.3 (65.1-65.6)		
Sex			p<0.001	
   Men	4004 (47)	63.5 (63.1-63.9)		
   Women	4554 (53)	67.2 (66.8-67.5)		
Men, by age			p<0.001	
   18-24 years	232 (5.8)	61.3 (59.4-63.1)		
   25-34 years	458 (11)	61.0 (59.9-62.1)		
   35-44 years	604 (15)	62.4 (61.4-63.4)		
   45-54 years	797 (20)	63.0 (62.2-63.9)		
   55-64 years	853 (21)	65.3 (64.6-66.0)		
   65-74 years	772 (19)	66.5 (65.7-67.2)		
   ≥ 75 years	288 (7.2)	67.6 (66.5-68.7)		
Women, by age			p<0.001	
   18-24 years	367 (8.1)	63.1 (61.8-64.4)		
   25-34 years	629 (14)	65.4 (64.5-66.3)		
   35-44 years	744 (16)	67.0 (66.1-67.9)		
   45-54 years	1000 (22)	66.8 (66.1-67.6)		
   55-64 years	927 (20)	68.7 (68.0-69.5)		
   65-74 years	716 (16)	69.5 (68.6-70.4)		
  ≥ 75 years	171 (3.8)	70.0 (68.5-71.5)		
Education (if age >25 years)			p<0.001	
   Low	3428 (40)	63.5 (63.1-63.9)2		
   Medium	1920 (22)	66.0 (65.5-66.5) 2		
   High	2507 (29)	68.6 (68.1-69.0) 2		
Perceived household economy 			p<0.001	
   Restricted	1249 (15)	61.5 (60.7-62.2) 2		
   Fair	2288 (28)	64.7 (64.2-65.2) 2		
   Good	4774 (57)	66.6 (66.2-66.9)2		
1The estimates are calculated using population weights weighing for county, age (5 categories) and sex in the the Norwegian Public Health Survey. 2p-values are calculated from linear regression, adjusting for covariates if relevant. 3 Estimates from linear regression adjusting for age and sex. CI: Confidence Interval	
Supplementary table S3
Table S3. Correlation matrix using pairwise Pearson's correlations between components in the Norwegian Dietary Guideline Index.
	Fruit	Vegetables	Wholegrain	Fish	Red meat	Salt	Fat for frying	Fat for bread	Cheese	Milk/yoghurt	Water	Sugary drinks	Chocolate/candy	Sweet pastries	Salty snacks	
Fruit	1.00												
Vegetables	0.39	1.00											
Wholegrain	0.25	0.25	1.00										
Fish	0.19	0.22	0.25	1.00										
Red meat	0.05	0.02	-0.05	0.11	1.00									
Salt	0.06	0.05	0.09	0.09	0.10	1.00									
Fat for frying	0.11	0.13	0.06	0.03	0.06	0.05	1.00								
Fat for bread	0.00	-0.03	0.06	0.01	0.01	0.05	0.12	1.00							
Cheese	-0.11	-0.10	-0.26	-0.04	0.11	0.01	-0.02	-0.01	1.00						
Milk/yoghurt	0.12	0.00	0.17	0.11	-0.05	0.02	-0.01	0.01	-0.02	1.00					
Water	0.18	0.25	0.17	0.12	0.02	0.03	0.07	-0.03	-0.07	0.00	1.00					
Sugary drinks	-0.04	0.13	0.12	0.12	0.10	0.05	0.04	0.03	0.00	0.00	0.14	1.00				
Chocolate/candy	-0.04	0.00	0.01	0.07	0.07	0.05	0.01	0.00	0.06	-0.01	0.05	0.14	1.00			
Sweet pastries	-0.09	0.01	-0.05	-0.06	0.03	0.00	0.03	0.01	0.07	-0.07	0.04	0.06	0.27	1.00		
Salty snacks	0.03	0.06	0.07	0.12	0.11	0.13	0.02	-0.01	0.00	0.01	0.05	0.14	0.29	0.10	1.00	
Total index score	0.59	0.53	0.58	0.46	0.17	0.34	0.32	0.19	-0.06	0.47	0.37	0.25	0.16	0.03	0.20	
Bold font indicates p<0.01.		


Supplementary table S4
Table S4. Eigenvalues and proportions in dimensions from principal component analysis in the Norwegian Dietary Guideline Index.
Dimension	Eigenvalue	Difference	Proportion	
Dimension 1	2.23	0.62	0.15	
Dimension 2	1.61	0.44	0.11	
Dimension 3	1.17	0.03	0.08	
Dimension 4	1.15	0.05	0.08	
Dimension 5	1.10	0.12	0.07	
Dimension 6	0.98	0.02	0.07	
Dimension 7	0.97	0.10	0.06	
Dimension 8	0.87	0.02	0.06	
Dimension 9	0.84	0.04	0.06	
Dimension 10	0.80	0.03	0.05	
Dimension 11	0.77	0.03	0.05	
Dimension 12	0.74	0.10	0.04	
Dimension 13	0.64	0.04	0.04	
Dimension 14	0.61	0.09	0.03	
Dimension 15	0.52			


Supplementary table S5
Table S5. Components in the Norwegian Dietary Guideline Index and corresponding loadings1 from principal component analyses in the first three dimensions.
Component	Dimension 1	Dimension 2	Dimension 3	
Fruit	0.41	-0.21	0.14	
Vegetables	0.44	-0.08	0.01	
Wholegrain	0.43	-0.17	-0.18	
Fish	0.38	0.02	0.08	
Red meat	0.10	0.29	0.48	
Salt	0.17	0.15	0.28	
Fat for frying	0.16	0.05	0.41	
Fat for bread	0.04	0.02	0.37	
Cheese	-0.19	0.27	0.35	
Milk/yoghurt	0.16	-0.17	-0.10	
Water	0.30	0.03	-0.17	
Sugary drinks	0.21	0.31	-0.05	
Chocolate/candy	0.10	0.52	-0.29	
Sweet pastries	-0.04	0.41	-0.25	
Salty snacks	0.20	0.42	-0.13	
1r loadings are correlation coefficients (r) between input variables (components) and extracted dimensions.
